# A Delicate Balance: Immunotherapy for Endometrial Cancer in a Patient With Multiple Sclerosis

**DOI:** 10.14740/jmc5260

**Published:** 2026-07-01

**Authors:** Antonio Ruggiero, Chiara Redemagni, Cristina Angela Camnasio, Vittoria Morteo, Federica Carcagni, Simone Zatti, Ilaria Strada, Marianna Roccio, Eleonora Rigoni, Chiara Cassani, Elena Colombo

**Affiliations:** a Department of Clinical, Surgical, Diagnostic and Pediatric Sciences, University of Pavia, Pavia, Italy; b Unit of Obstetrics and Gynecology, IRCCS S. Matteo Foundation, Pavia, Italy; c Multiple Sclerosis Centre, IRCCS Mondino Foundation, Pavia, Italy; d These authors contributed equally to this article.; e These authors share the co-last authorship.

**Keywords:** Endometrial cancer, Multiple sclerosis, Immune checkpoint inhibitors, Dostarlimab, Disease modifying therapies

## Abstract

Multiple sclerosis (MS) is a chronic immune-mediated disease that may complicate oncological management, particularly when immune checkpoint inhibitors (ICIs) are indicated. Evidence regarding the safety of ICIs in patients with pre-existing MS remains limited, especially in older patients and in the first-line treatment of endometrial cancer (EC). We present the case of a 71-year-old woman with relapsing–remitting MS (radiologically stable for > 12 months) and stage IIIC1 mismatch repair–deficient/microsatellite instability–high EC who was treated with first-line carboplatin–paclitaxel combined with programmed cell death protein 1 (PD-1) inhibitor dostarlimab. Ozanimod, which had been used to manage her MS, was temporarily discontinued prior to chemotherapy. No clinical relapses or new magnetic resonance imaging (MRI) activity were observed during combination therapy or throughout a 12-month interim follow-up. Dostarlimab maintenance therapy is ongoing. This case suggests that ICIs may be cautiously integrated into the treatment of selected patients with MS and advanced EC when baseline neurological stability, patient age, and prior disease-modifying therapy are carefully considered. Close interdisciplinary collaboration and structured neurological monitoring are essential. Longer follow-up is required to confirm long-term neurological safety.

## Introduction

Multiple sclerosis (MS) is a chronic autoimmune disorder characterized by inflammation and neurodegeneration within the central nervous system (CNS), leading to neurological symptoms such as visual disturbances, motor and sensory deficits, and cognitive impairment [1]. The etiology of MS is multifactorial, involving genetic predisposition and environmental triggers [2].

Disease-modifying therapies (DMTs) represent the foundation of MS management, reducing inflammatory disease activity and delaying disability progression. They work by suppressing or modulating the immune system with different degrees of efficacy and safety [3].

As the population ages, the number of older adults living with MS continues to rise, largely due to earlier diagnoses and improved survival rates. In older patients, the risk–benefit profile of long-term DMT exposure becomes less favorable, owing to immunosenescence, comorbidities, and increased susceptibility to adverse events [4]. Among various comorbidities, cancer is especially prevalent in individuals with MS. Even though safety results from clinical trials exploring associations between specific DMTs and cancer risk were reassuring, several studies reported possible weak signals of association. This may be attributed to both a dysregulated immune response underlying MS and prolonged exposure to DMTs, which can influence T-cell function. Nonetheless, the coexistence of MS and cancer is becoming increasingly common, underscoring the need for further research into the long-term safety of MS treatments [5]. An additional layer of complexity emerges when patients with MS undergo immune checkpoint inhibitors (ICIs) as part of oncological treatment. ICIs, including programmed cell death protein 1 (PD-1) inhibitors such as dostarlimab, have revolutionized cancer therapy by harnessing the immune system to target malignancies. However, their use in patients with autoimmune diseases poses significant challenges. The immunostimulatory effects of ICIs can exacerbate autoimmune activity, leading to the potential for MS relapses or other immune-mediated adverse events [6].

Managing DMTs in this context requires a delicate balance to mitigate autoimmune activation without compromising the effectiveness of cancer immunotherapy. Emerging evidence suggests that some patients with MS can tolerate ICIs with careful monitoring and prompt intervention for adverse effects. In the largest multicentric study to date, Androdias et al [7] included 18 MS patients treated with ICIs for various cancers and found that only 17% experienced MS activity, primarily younger patients who had discontinued their DMTs. These findings suggest that ICIs may be used safely in many patients with MS, provided there is careful neurological monitoring and interdisciplinary collaboration between oncologists and neurologists [8].

Endometrial cancer (EC) is the most common gynecological malignancy in high-income countries, with rising incidence in older women.

Molecular classification has transformed EC management, identifying mismatch repair–deficient (dMMR) and microsatellite instability–high (MSI-H) tumors as particularly responsive to immune checkpoint inhibition. The RUBY trial established the combination of dostarlimab with carboplatin–paclitaxel as a new standard of care for advanced or recurrent dMMR/MSI-H EC.

However, the use of ICIs in patients with pre-existing autoimmune diseases, including MS, raises concerns regarding immune-mediated disease reactivation. Evidence remains limited to retrospective cohorts and case reports, and data on optimal DMT management during ICI therapy are scarce. This uncertainty is particularly relevant during prolonged ICI maintenance phases.

Here, we describe a case of advanced MSI-H EC treated with first-line dostarlimab-based chemo-immunotherapy in a patient with stable MS, focusing on interdisciplinary management, neurological monitoring, and decision-making regarding DMT discontinuation and maintenance immunotherapy.

## Case Report

A 71-year-old woman with relapsing–remitting MS (RRMS) presented with abnormal vaginal bleeding. Her MS history was notable for mild disease, with a baseline Expanded Disability Status Scale (EDSS) score of 1.5, no clinical relapses for several years, and radiological stability on brain and spinal magnetic resonance imaging (MRI). Her past medical history included hypertension, atrial fibrillation, and a history of myocardial infarction. The patient’s MS was well-controlled with ozanimod, an orally administered sphingosine-1-phosphate receptor modulator (S1PRM), and she had no previous history of malignancy. Ozanimod was started 1 year before, due to persistence of radiological activity on a first-line treatment, dimethyl fumarate. Upon initial evaluation, pelvic ultrasound revealed a heterogeneous endometrium with suspected infiltration into the myometrium. The lesion appeared to extend to within a few millimeters of the serosa on the anterior uterine wall and seemed to surpass the internal cervical os. Subsequent hysteroscopic examination showed that the uterine anterior wall was occupied by friable, highly vascularized tissue, which was seen to obliterate the left tubal ostium and extend near the internal cervical os. Multiple biopsies of the lesion were taken using grasping forceps. MRI of the lower abdomen, both with and without contrast, revealed a large endometrial mass infiltrating the outer half of the myometrium on the left side of the uterine fundus. However, no obvious signs of invasion into surrounding periuterine tissues were noted. Staging computed tomography of the chest and abdomen revealed no significant abnormalities outside the uterus.

A biopsy revealed low-grade (G2) endometrioid EC, while immunohistochemical staining for mismatch repair (MMR) proteins detected the loss of MSH6 expression. Based on these diagnostic findings, the patient subsequently underwent laparoscopic surgery, which included a total hysterectomy, bilateral salpingo-oophorectomy, and bilateral sentinel lymph node biopsy. Histopathological examination of surgical specimens confirmed the presence of stage IIIC1 EC according to International Federation of Gynecology and Obstetrics (FIGO) criteria because of the positivity of the lymph node retrieved. The high-risk features of the diseases combined with the loss of MSH6 gene expression indicated a need for a comprehensive oncological approach. The carcinoma showed myometrial invasion, affecting more than 50% of the myometrial thickness, with an invasion depth of approximately 12 of 14 mm. The microcystic, elongated, and fragmented (MELF) pattern of invasion was present, though no serosal involvement was observed. Focal lymphovascular invasion was noted, while there was no involvement of the lower uterine segment, cervix, fallopian tubes, ovaries, or parametrial tissues. Molecular analysis showed the presence of likely pathogenic mutations in PTEN, pathogenic mutations in PIK3CA, and a variant of uncertain significance (VUS) in the RET gene, along with an MSI-H. Additionally, POLE and TP53 were found to be negative.

Prior to chemotherapy initiation, the patient underwent a brain and spinal cord MRI to assess for MS activity, which was stable. Following the MRI, consultation with her treating neurologist led to the decision to suspend her ozanimod treatment before chemotherapy. In light of the scarcity of data regarding ICIs use in patients affected by MS, a close neurological follow-up with clinical evaluation and MRI every 6 months has been established. The patient began treatment with six cycles of chemotherapy consisting of carboplatin (AUC5), paclitaxel (175 mg/m^2^), and dostarlimab (500 mg), which was introduced from the second cycle according to the expanded access protocol (EAP). During the treatment, the patient experienced several toxicities related to chemotherapy rather than immunotoxicity. A grade 2 neutropenia and grade 2 diarrhea according to Common Terminology Criteria for Adverse Events (CTCAE) occurred after the second cycle, leading to the suspension of chemotherapy for 1 week to allow for recovery. During the therapy, the patient also experienced a taxol-related neurotoxicity, initially presenting as grade 2 paresthesia (CTCAE) in the distal extremities of both upper and lower limbs. Treatment with supplementation based on alpha-lipoic acid led to a slight improvement. The symptoms currently persist as grade 1 paresthesia, with gradual and modest improvement over time. Despite these challenges, the patient continued her treatment regimen with close monitoring of her clinical status. No radiological or clinical signs of MS activity or progression were seen during the treatment. At the conclusion of chemotherapy, the patient initiated maintenance therapy with dostarlimab 1,000 mg every 6 weeks, following the protocol of the RUBY clinical trial (NCT03981796). This maintenance regimen aims to prolong progression-free survival and improve overall survival in patients with advanced or recurrent EC. The patient is currently continuing dostarlimab therapy, which is planned for a duration of 3 years. She is currently on the 12th cycle of maintenance with immunotherapy. Regular follow-up care is provided to monitor for any signs of EC and MS recurrence or progression. The patient underwent chest and abdominal computed tomography (CT) scans for oncological disease reassessment at the end of six cycles of chemotherapy and 6 months after the initiation of maintenance therapy, showing no evidence of disease recurrence. As planned, spinal cord and brain MRI were performed at 6 and 12 months after initiation of therapy, demonstrating no signs of MS relapse.

Given the retrospective nature of the case report, Institutional Review Board approval was waived. The study was performed in accordance with the Declaration of Helsinki.

## Discussion

The management of MS in complex clinical scenarios, such as the coexistence of other autoimmune conditions and cancer, requires individualized planning and a multidisciplinary approach. This case highlights the challenges involved in managing a patient with MS and stage IIIC1 EC, emphasizing the balance between effective oncological treatment and neurological stability.

Recent advancements in molecular profiling and therapeutic strategies have enabled tailored and effective treatment approaches for EC. In 2023, the Food and Drug Administration (FDA) approved the combination of dostarlimab with carboplatin/paclitaxel for advanced EC characterized by mismatch repair deficiency (dMMR) and/or MSI-H, based on survival data from the phase 3 RUBY trial (ENGOT-EN6-NSGO/GOG3031). This combination is now the standard of care for first-line treatment of advanced or recurrent EC [9].

The patient was undergoing treatment with ozanimod, a second-generation S1PRM. S1PRMs inhibit the migration of lymphocytes from lymphoid tissues into peripheral blood, potentially preventing their entry into the CNS. The most common adverse events during S1PRM treatment are infections, related to drug-induced lymphopenia. The decision to stop ozanimod was taken, according to the technical data sheet, to avoid excessive immunosuppression.

Nonetheless, the discontinuation of S1PRMs, especially fingolimod, can carry a risk of rebound, defined as severe disease worsening exceeding baseline activity after treatment cessation [10]. Baseline investigations, including laboratory tests, imaging studies (MRI with T2-weighted and gadolinium-enhanced sequences), and regular neurological evaluations, play a crucial role in monitoring disease progression and ensuring the safety of treatment decisions. In this case, the decision to withdraw ozanimod was made considering the imminent start of chemotherapy and the pharmacodynamics of ozanimod, which has sustained effects for at least 1 month after its discontinuation. Ozanimod, in fact, is a prodrug whose main active metabolite, CC-112273, has a considerably long half-life of approximately 264 h (about 11 days). After discontinuing ozanimod, the median time for peripheral blood lymphocytes to return to the normal range is approximately 30 days. The short washout period prior to the first dose of chemotherapy was crucial to avoid disease breakthroughs, and close monitoring could prevent potential disease reactivation during immunotherapy.

In the largest multicentric study to date, Androdias et al [7] reported that only three patients (17%) experienced MS activity, all of whom had recently discontinued DMTs and were under 50 years old. In contrast, the majority of patients, especially older ones, remained stable despite receiving ICIs. These findings suggest that patient age, baseline disease activity, and DMT management are critical factors, highlighting the importance of individualized therapeutic decisions and close interdisciplinary collaboration between neurologists and oncologists [7].

Moreover, this case provides an example of how a multidisciplinary approach can optimize patient outcomes in complex scenarios. Collaboration between oncologists, neurologists, and other healthcare professionals facilitated individualized management, balancing the aggressiveness of cancer therapy with the need to preserve neurological stability. The experience gained underscores the value of proactive monitoring, early recognition of therapy-related adverse effects, and appropriate supportive measures.

This case serves as a practical reference for managing patients with concurrent autoimmune and oncological conditions, illustrating strategies to safely navigate potential treatment conflicts. It reinforces that continuous evaluation and personalized adjustments to therapy are essential to achieve both oncological efficacy and neurological safety.

The findings from Androdias et al [7], together with our patient’s experience, support the notion that ICIs can be safely administered in selected MS patients under close neurological monitoring, even after DMT discontinuation. Patient age, disease activity, and prior treatment history should guide individualized decisions. Overall, this case emphasizes the critical role of proactive planning, vigilant monitoring, and collaborative care in optimizing outcomes for patients with overlapping autoimmune and oncological conditions.

A critical unresolved issue concerns DMT management during prolonged ICI maintenance. In this case, continued suspension of ozanimod was favored due to sustained MS stability and concern for compounded immunosuppression. Reintroduction of immunomodulatory therapy could be considered if oncologic disease remains controlled and MS activity re-emerges, ideally selecting agents with limited systemic immunosuppressive effects. An MS flare during maintenance ICI would necessitate temporary immunotherapy interruption, corticosteroid treatment, and reassessment within a multidisciplinary framework.

Table 1 [7, 8] summarizes published cases of ICI use in MS, highlighting heterogeneous management strategies and generally favorable neurological outcomes.

**Table 1:** Published Experience of Immune Checkpoint Inhibitors in Patients With MS

Study	Cancer type	ICI	MS status	DMT management	Neurological outcome
Androdias et al, 2024 [7]	Various	Anti–PD-1/PD-L1	RRMS/SPMS	DMT discontinued in most	17% MS activity, mainly younger patients
Gelipter et al, 2024 [8]	Various	Mixed ICIs	Mixed	Variable	Low relapse rate overall
Present case	Endometrial cancer	Dostarlimab	RRMS	Ozanimod discontinued	No relapse or MRI activity at 12 months

Overview of selected published reports and cohorts describing ICI use in patients with MS. MS: multiple sclerosis; DMTs: disease-modifying therapies; ICIs: immune checkpoint inhibitors; PD-1: programmed cell death protein 1; PD-L1: programmed death-ligand 1; RRMS: relapsing-remitting MS; SPMS: secondary progressive MS; MRI: magnetic resonance imaging.

**Figure 1:**
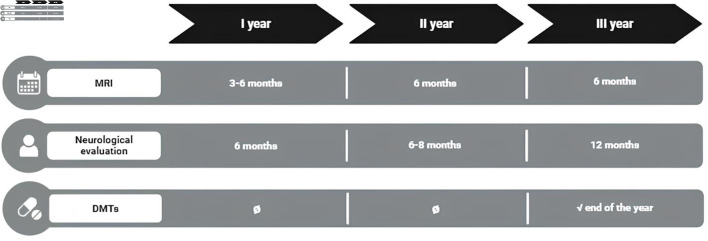
Longitudinal multidisciplinary management timeline. The figure illustrates the clinical timeline of oncological treatment, neurological monitoring (clinical examination and MRI at baseline, 6 and 12 months), and decision points regarding disease-modifying therapy management. The monitoring strategy was based on available observational evidence and expert consensus rather than formal guidelines. DMTs: disease-modifying therapies; MRI: magnetic resonance imaging.

Figure 1 outlines the rationale-driven monitoring strategy applied in our patient, based on existing observational evidence and expert consensus.

### Conclusions

This case supports the cautious use of ICIs in selected patients with stable MS and advanced EC. Baseline neurological stability, advanced age, and structured monitoring were central to the favorable outcome observed. Our findings should be interpreted as hypothesis-generating, and longer follow-up is needed to define long-term safety during extended maintenance immunotherapy. Close interdisciplinary collaboration remains essential.

## Data Availability

The data that support the findings of this study are available from the corresponding author upon reasonable request.

## Abbreviations

MS: multiple sclerosis
CNS: central nervous system
DMTs: disease-modifying therapies
ICIs: immune checkpoint inhibitors
EC: endometrial cancer
CTCAE: Common Terminology Criteria for Adverse Events
S1PRM: sphingosine-1-phosphate receptor modulator
RRMS: relapsing-remitting MS
MRI: magnetic resonance imaging
FIGO: International Federation of Gynecology and Obstetrics
dMMR: mismatch repair deficiency
